# A Case Report of a Ventral Hernia Containing a Liver Cyst in a Patient with Autosomal Dominant Polycystic Kidney Disease

**DOI:** 10.7759/cureus.6573

**Published:** 2020-01-05

**Authors:** Mridul Pansari, Robert D Rawlinson, David Rubay, Thomas Genuit, Andrew Ross

**Affiliations:** 1 Surgery, Charles E. Schmidt College of Medicine, Florida Atlantic University, Boca Raton, USA

**Keywords:** poly-cystic kidney disease, ventral hernia, cystic liver

## Abstract

Autosomal dominant polycystic kidney disease (ADPKD) is the most commonly inherited renal disorder and the fourth most common cause of end-stage renal disease. ADPKD is a systemic disease with multiple extrarenal manifestations, including cystic involvement of other organs, such as the liver and pancreas, and connective tissue abnormalities. The prevalence of hernias is higher in patients with ADPKD. It has been hypothesized that these hernias are the result of abnormal extracellular matrix production and/or increased intra-abdominal pressure from the cyst burden. We present a case of a 56-year-old female with polycystic kidney disease who was admitted for an incarcerated ventral hernia. The patient presented with obstructive symptoms concerning for bowel impingement. The patient underwent operative management, and during the procedure, an incarcerated liver cyst was identified in the hernia sac. This was successfully reduced, and the hernia was repaired with mesh.

## Introduction

Autosomal dominant polycystic kidney disease (ADPKD) is a systemic disease with multiple extrarenal manifestations, including cystic involvement of other organs, such as the liver and pancreas, and connective tissue abnormalities [1-2]. Liver cysts are the most common extrarenal manifestation of ADPKD, and up to 94% of patients with ADPKD older than 35 years of age may demonstrate such cysts in the liver. The prevalence of abdominal wall hernias is also higher in patients with ADPKD. It has been hypothesized that these patients develop hernias as a result of abnormal extracellular matrix production and/or increased intra-abdominal pressure from the cyst burden [3]. One retrospective review demonstrated that 45% of patients with ADPKD-related end-stage renal disease developed hernias, as compared with 16% in age and gender-matched patients with renal failure due to other etiologies, and only 4% in general surgical control subjects without renal failure [3]. A review of the literature on ADPKD did not reveal any prior publications that describe a hernia containing an incarcerated liver cyst.

## Case presentation

**Figure 1:**
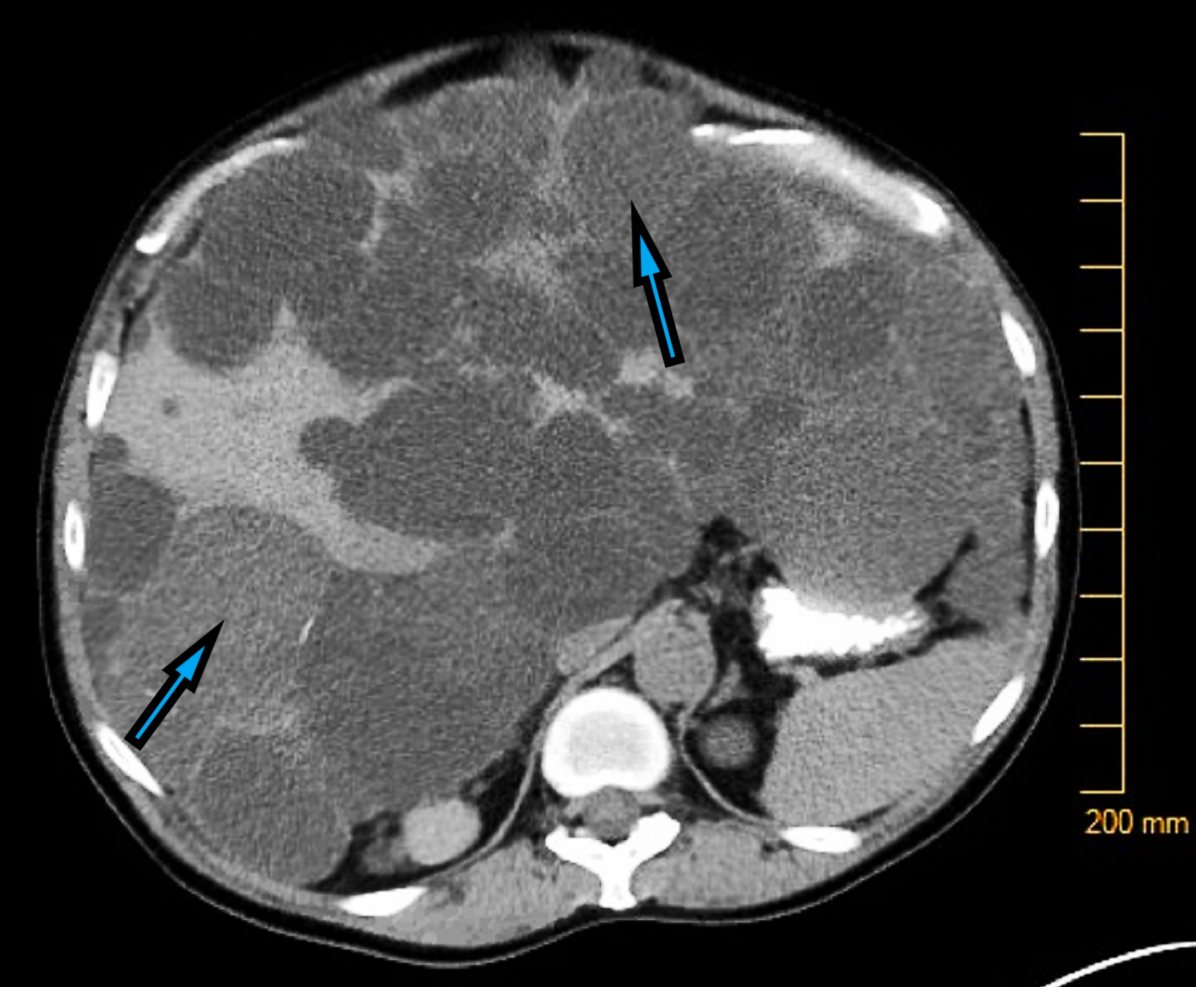
Numerous liver and kidney cysts in a patient with autosomal dominant polycystic kidney disease (ADPKD)

A 56-year-old female with a past medical history of ADPKD with associated polycystic liver disease (PCLD) presented to the emergency department with a one-day history of acute onset diffuse crampy abdominal pain associated with nausea and vomiting. On presentation, the patient was afebrile and hemodynamically stable. Physical examination revealed a distended abdomen, marked nodular hepatomegaly, a firm, nodular non-reducible ventral bulge in the epigastrium, and a small reducible umbilical hernia. Laboratory workup, including a complete blood count and comprehensive metabolic panel, was unremarkable (Figure 1).

**Figure 2:**
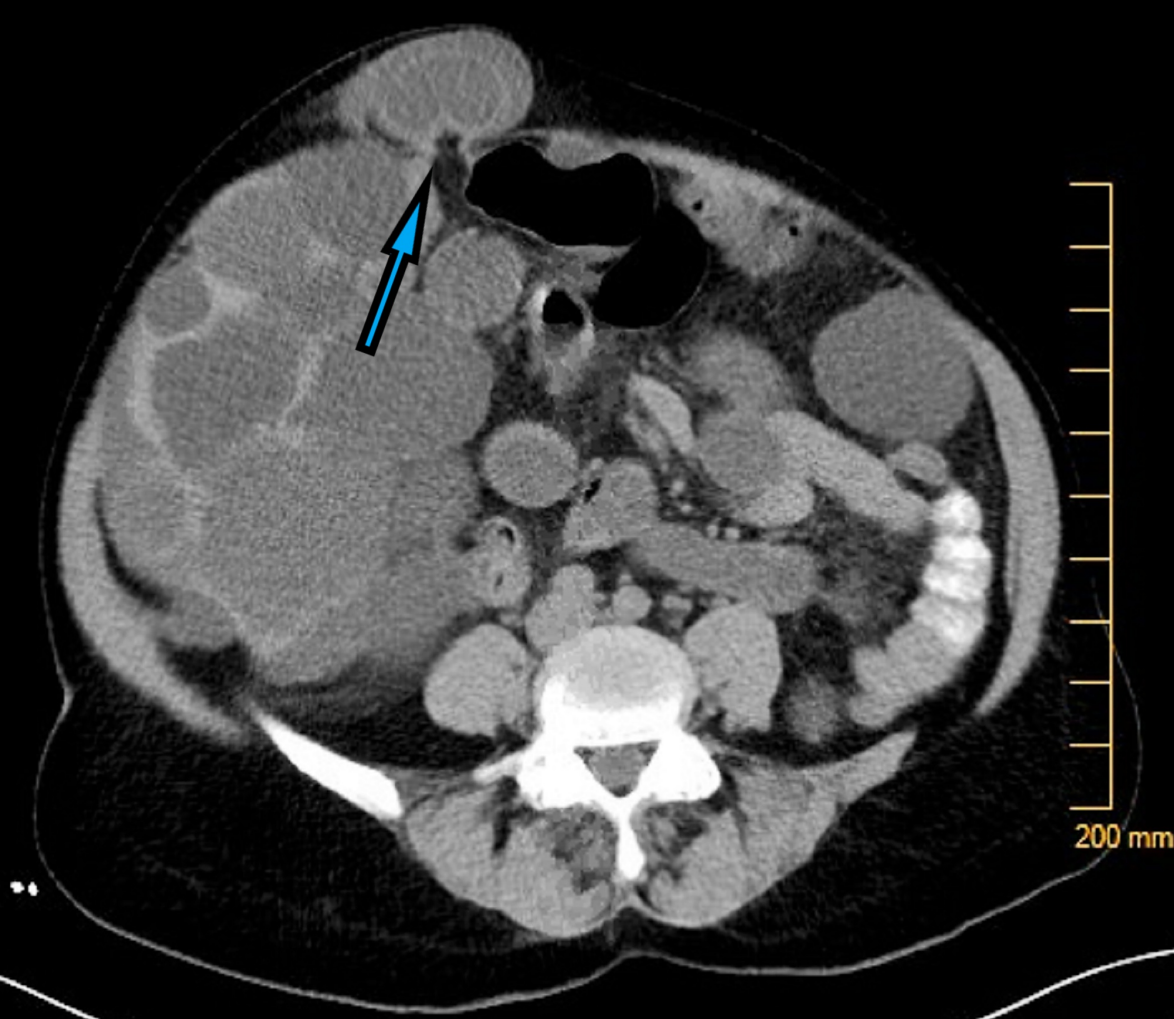
Right paramedian epigastric ventral hernia with a narrow hernia defect, containing a rim-enhancing, fluid-filled structure raising the concern for bowel incarceration

The patient underwent computed tomography (CT) of the abdomen and pelvis with intravenous and oral contrast, which demonstrated an epigastric ventral hernia containing a rim-enhancing fluid-filled structure and a narrow hernia defect, possibly representing an incarcerated loop of bowel (Figure 2).

Given the patient’s symptoms and imaging results, the patient underwent urgent surgical intervention (open hernia repair). During the procedure, an incarcerated liver cyst was identified in the hernia sac. This was successfully reduced. There was no evidence of bowel involvement in the hernia. The inspection of the abdomen revealed no other evidence of intestinal obstruction. The hernia defect was repaired using a self-expanding polypropylene, and ePTFE patch (BD Bard Ventralex™; CR Bard Inc., Murray Hill, NJ) mesh in an underlay fashion with circumferential sutures and closing the fascia over the mesh. The patient’s postoperative course and recovery were unremarkable; she was advanced to a regular diet and was discharged on a postoperative day one. Two-week outpatient follows up evaluation noted resolution of her hernia and no recurrence of her symptoms.

## Discussion

ADPKD is the most commonly inherited kidney disease and is associated with cystic manifestations in other organs such as the liver and pancreas. PCLD is the most common extrarenal manifestation of ADPKD, noted in up to 90% of patients. Cyst burden (cyst number and volume) tend to increases with age, and there is a corresponding 0.9% to 3.2% increase in liver size per year [4-5]. Mutations in two genes, PKD1 and PKD2, are associated with ADPKD [4-5]. PCLD secondary to ADPKD should not be confused with isolated PCLD, which also is inherited in an autosomal dominant manner and is related to mutations the PRKCSH and Sec 63 genes [6]. When compared to a patient with ADPKD, PCLD patients with isolated PCLD tend to have a greater number of liver cysts that are larger [7]. Regardless of the cyst burden, these patients are unlikely to develop end-stage liver failure or require liver transplantation, in the absence of kidney cysts and renal dysfunction [7].

In ADPKD related PCLD, the degree of cystic liver involvement is highly variable. The prevalence and cyst burden is usually greater in females [8]. Smaller cysts are typically asymptomatic, and most symptoms are the result of mechanical compression of nearby structures or complications within the cysts themselves [9-10]. Abdominal distention, early satiety, nausea with or without emesis, esophageal reflux, dyspnea, and lower back pain are all common compressive symptoms. Symptoms related to compression of the hepatic veins (Budd-Chiari syndrome), inferior vena cava, portal vein, and bile ducts can occur. Complications from these cysts include intracystic hemorrhage, infection, torsion, traumatic rupture, and rarely cystadenoma or cystadenocarcinoma [11]. The liver parenchymal volume and synthetic function are usually preserved even in more extensive cystic disease [12]. Treatment is usually aimed at palliating symptoms and reducing liver volume. Medical therapy includes somatostatin analogs and sirolimus (rapamycin). Somatostatin analogs are thought to work by inhibiting cyclic adenosine monophosphate, a major promoter of hepatic cyst growth, by means of increasing chloride and bicarbonate transport of fluid secretion across the apical membrane of cholangiocytes [13-14]. Sirolimus and other mTOR (also known as mammalian target of rapamycin, FK506-binding protein, or 12-rapamycin-associated protein 1 (FRAP1)) inhibitors are thought to have an antiproliferative effect. 

Surgical treatment may include cyst aspiration and sclerotherapy for one or a few dominant cysts. Open or laparoscopic cysts fenestration has been tried, but may lead to complications such as ascites, pleural effusion, hemorrhage, and biliary leakage [15]. Partial hepatectomy can be considered in patients with massive hepatomegaly, but careful planning must assure that sufficient hepatic parenchyma is left in place. Transcatheter arterial embolization may be appropriate for patients who cannot tolerate surgical intervention [16], and liver transplantation remains the most definitive treatment for PCLD. When transplantation is considered, the combined liver-kidney transplant should be considered for patients with concomitant polycystic kidney disease that have evidence of renal insufficiency.

The increased prevalence of hernias in patients with ADPKD may be the result of abnormal extracellular matrix production and/or increased intra-abdominal pressure from organomegaly (cyst burden) [3]. These patients may present with inguinal hernias (13% versus 4% in unaffected relatives) and umbilical hernias (7% versus 2%) [1]. A retrospective review on 85 patients with ADPKD and end-stage renal disease demonstrated that 45% (38 or 85 patients) developed hernias as compared with 16% of age and gender-matched patients with renal failure from other causes, and only 4% of similar non-renal failure, non-cystic kidney control subjects [3]. Hernias are an especially important concern in patients with ADPKD on peritoneal dialysis. In one small retrospective study, 46% (6 of 13) of male patients with ADPKD on peritoneal dialysis developed inguinal hernias compared to only 3% (1 of 30) of non-ADPKD males on peritoneal dialysis [17]. To date, there have been no reports of liver cyst herniation through a ventral abdominal wall defect. There are no definitive guidelines or recommendations for what type of hernia repair should be used in these cases. It can be argued that the underlying matrix or soft tissue defect should lead the surgeon away from simple primary repair, even in relatively small defects, and to consider utilization of a mesh.

## Conclusions

Patients with ADPKD are very likely to experience PCLD. These patients also have a significantly increased risk of developing abdominal wall hernias. In this case, the patient’s epigastric ventral hernia contained a liver cyst that was not ruptured and successfully reduced. There are no definitive guidelines on how to address ischemic, infected or ruptured cysts. Cyst fenestration or resection may lead to complications, including ascites formation, bleeding or bile leak. There are also no definitive guidelines for the optimal repair of the hernia defect in ADPKD, but the underlying matrix or soft tissue defect should lead to consideration of mesh augmentation of the repair, even in relatively small defects. Future studies would be needed to address these questions.
